# A Vision For the Future of Cardiovascular Medicine Practise in Ghana: Inspiration From the Yale-New Haven Health System

**DOI:** 10.5334/gh.1357

**Published:** 2024-09-13

**Authors:** Kofi Tekyi Asamoah, Michael Harry Beasley

**Affiliations:** 1 National Cardiothoracic Centre, Korle-Bu Teaching Hospital, Accra, Ghana; 2 Department of Medicine, Korle-Bu Teaching Hospital, Accra, Ghana; 3 Advanced Heart Failure and Transplant Cardiology, Yale-New Haven Health Heart and Vascular Center, New Haven, Connecticut, United States of America

**Keywords:** Ghana, cardiovascular, global health, task shifting

## Abstract

The Ghana Physicians and Surgeons Foundation (GPSF) of North America sponsors Ghanaian clinical fellows to undertake an eight-weeklong clinical observation with the Yale University School of Medicine and Yale-New Haven Health (YNHH) annually, through the Residents in Training Educational Stipend (RITES) programme. This offers the opportunity to appreciate new perspectives in clinical care to improve Ghana’s healthcare standard.

The cardiovascular medicine workforce at the YNHH is heterogenous, with significant reliance on non-doctor cadres of health workers who demonstrate competence. This is contrasted from the Ghanaian system which despite having a poorer physician-patient ratio, is heavily dependent on doctors. Technological advancements are minimal in Ghana, posing diagnostic and therapeutic challenges which are otherwise minimised at the YNHH. A strong patient-centred culture, coupled with a coordinated emergency response system that ensures appropriate timely transfers, culminate in good care and outcomes.

Ideas on how the experience can be translated to Ghanaian clinical practise in cardiovascular medicine, after participating in the RITES programme, are shared in this paper with an emphasis on task sharing, strengthening emergency response systems and improving technological sophistication through capacity building, mentorship and improved health financing.

## Introduction

The Residents in Training Educational Stipend (RITES) programme of the Ghana Physicians and Surgeons Foundation of North America allows Ghanaian clinical fellows to spend eight weeks in an enriching clinical experience at Yale-New Haven Health (YNHH), with the aim to improve local care in Ghana. Following participation in this programme, the experience is discussed and related to the Ghanaian cardiovascular healthcare system.

### The YNHH experience

The cardiovascular medicine workforce of the YNHH comprised physician assistants (PAs), nurse practitioners (NPs), and doctors. All these cadres of staff were equally responsible for the day-to-day care of patients on admission, under the supervision of attending physicians. They reviewed their patients independently and drew management plans for discussion during ward rounds. Intriguingly, treatment plans of NPs and PAs needed little modification, while the depth of clinical discussions demonstrated an understanding of management principles. Nurse practitioners and PAs also successfully led emergency resuscitation attempts on the wards, without the need for interference by the attending physicians present. All cadres responded to referrals, performed pre-discharge counselling, and ran clinics.

There was an ST-elevation myocardial infarction (STEMI) activation service available to paramedics and hospital staff with suspected cases, manned by a clinical fellow. Once activated, the fellow immediately reported to the ward or emergency unit (for out-of-hospital activations) regardless of other duties and waited for the patient. On patient arrival, staff played their individual roles without clashes. Once STEMI was confirmed, the patient was sent to the cardiac catheterization laboratory within 30 minutes for revascularization therapy.

While theoretical knowledge was comparable with what pertains in Ghana, there was a gulf between available logistics in both settings. Patients with heart failure and shock had treatments titrated to real-time measurements of intracardiac pressures, measured with invasive devices like CardioMEMS® and Swan-Ganz catheters. Patients in shock also had access to mechanical circulatory support as needed. Cardiac telemetry was available for at-risk inpatients.

There were smooth transfers between sub-units for procedures like cardiac catheterizations and cardioversions, unaffected by time of day or shift changes. This was facilitated by telephone communication between teams in addition to formal consult requests. The smoothness of these transitions depicted a strong culture of teamwork and mutual respect. This culture is due to the hospital’s efforts to reinforce patient-centred care behaviours, with reminders posted at vantage points and broadcast on screens throughout the hospital.

### The cardiovascular medicine workforce in Ghana

Ghana had less than thirty actively practicing Cardiologists and less than twenty fellows in training at the time of writing.

Doctors bear the burden of care for patients with cardiovascular diseases. Cardiologists and Internists are primarily in urban centres, while medical officers (pre-residency) and PAs (who may operate unsupervised as there are few doctors) frequently address patient needs in semi-urban and rural settings. Some, however, practise in urban primary care centres. While Internists receive formal training in managing cardiovascular diseases during residency, medical officers and PAs need to deliberately seek continuous professional development programmes in cardiology. This influences their proficiency in recognising and managing cardiovascular diseases (1).

Furthermore, there is no integrated emergency response service that responds to home calls. If emergency services do transport such patients, there are no established channels of communication with specialised tertiary services. Such patients are, therefore, sent to general emergency units, where they go through routine assessments and are consequently handed over to internal medicine, on-call teams who then invite cardiology if necessary. This results in delays in receiving emergency cardiovascular care where needed.

### Lessons from YNHH – Nurses and physician assistants

A task shifting approach, akin to the YNHH model, will help improve the reach and quality of care (2). Ghana has PAs and nurses at various levels of care. Upgrading their knowledge and skills will allow them to practise aspects of cardiovascular care at an acceptable level of safety (2). The feasibility of such a model has been demonstrated on a smaller scale in Ghana (3), Cameroon (4), and Kenya (5).

### Lessons from YNHH – Doctors

Cardiovascular medicine in Ghana has a huge growth potential. While it is impractical to expect the same level of sub-specialisation as in high-income countries in the short-term, it is feasible to target improving general and emergency cardiology services within the next few years through optimising current care standards and improving staff numbers with trained non-doctor cadres.

Internists can supervise non-specialist and non-doctor cadres of staff at regional levels, consulting with fellows and consultants in tertiary centres where needed. There should be formal avenues for remote support to facilitate such consultations. The fellows are also vital in establishing communication channels with colleagues in other specialties to improve multidisciplinary care.

A robust cardiology fellowship programme with exposure to research and protected teaching and research time is vital, as it equips fellows with the requisite skills and knowledge to offer appropriate evidence-based care. The testimony of fellows is critical in encouraging residents to choose cardiology, improving the number of cardiologists rendering services in the country. On completion of fellowship, new cardiologists can support local capacity building through education, especially those from less urban areas.

As more cardiologists are produced over the next few years, advocacy for sub-specialised cardiology practise can be prioritized. With the current turnover of fellows into cardiologists, Ghana should have at least thirty new cardiologists within a decade, barring emigration. These can be sponsored to train further to develop the skills in performing and interpreting advanced tests and procedures for cardiac patients.

### Lessons from YNHH – Emergency services

A reliable emergency service that can communicate with the relevant specialists for timely interventions is required to improve outcomes for cardiovascular emergencies. This requires training paramedics in identifying these emergencies (including performing electrocardiograms for acute chest pain) and offering appropriate first aid. Paramedics must also be well-versed in resuscitation, including cardiac and non-cardiac arrest care pathways. Active helplines should be available round-the-clock for prompt responses to emergencies. This will reduce the time delays involved in care for such patients.

### How can this be realised?

Investments are needed through sponsorships for higher education and training, as well as procurement of modern logistics for clinical practise to allow for the application of newly obtained skills. Employment of more staff to allow for specialisation and training in patient-centeredness is also important. Remuneration must be improved to match the output desired from the ‘upgraded’ PAs, NPs, and doctors who develop added skills to offer the highest standard of care.

To ensure accessibility to patients, a robust health financing system is imperative. This reduces delays related to paying deposits before receiving care. Importation of logistics will also greatly benefit from tax reliefs and other government-based subsidies, ultimately lowering cost of procedures.

However, a possible threat to this model is that in acquiring these skills, other cadres may perceive themselves as equal to the physician and fail to submit to the leadership of the cardiologist. This could pose a threat to patient safety. The roles of each cadre must be codified. While PAs and NPs may acquire new skills and knowledge, physicians invest more time into training and research and are responsible for all patients within the team. Therefore, physicians must remain the highest authority within the team and must approve care decisions.

## Conclusion

Improving cardiovascular care in Ghana requires patience and mobilising various healthcare cadres already existing in the Ghanaian healthcare system. This transformation will require investment in human resource development, with improvements in remuneration and institutional structure.
